# Supplementary material for: Epidemiology of Hand, Foot, and Mouth Disease and Genetic Evolutionary Characteristics of Coxsackievirus A10 in Taiyuan City, Shanxi Province from 2016 to 2020
Source: Viruses. 2023 Mar 7;15(3):694. doi: 10.3390/v15030694 (PMC10052898; doi:10.3390/v15030694)
Supplement: Supplementary file 1 [file viruses-15-00694-s001.zip › viruses-2256317-supplementary.pdf]

Supplementary Table S1. GenBank accession numbers assigned for all the CVA10-Taiyuan sequences based on entire VP1 region sequenced in this study.

Coxsackievirus A10 (VP1)			
Accession Number	Strain	Accession Number	Strain
OP244619	TY2016022-SX-CHN-CVA10	OP244635	TY2019008-SX-CHN-CVA10
OP244620	TY2016033-SX-CHN-CVA10	OP244636	TY2019031-SX-CHN-CVA10
OP244621	TY2016080-SX-CHN-CVA10	OP244637	TY2019032-SX-CHN-CVA10
OP244622	TY2016140-SX-CHN-CVA10	OP244638	TY2019148-SX-CHN-CVA10
OP244623	TY2016143-SX-CHN-CVA10	OP244639	TY2019191-SX-CHN-CVA10
OP244624	TY2016188-SX-CHN-CVA10	OP244640	TY2019243-SX-CHN-CVA10
OP244625	TY2016196-SX-CHN-CVA10	OP244641	TY2019257-SX-CHN-CVA10
OP244626	TY2016198-SX-CHN-CVA10	OP244642	TY2019363-SX-CHN-CVA10
OP244627	TY2016199-SX-CHN-CVA10	OP244643	TY2019393-SX-CHN-CVA10
OP244628	TY2016213-SX-CHN-CVA10	OP244644	TY2019434-SX-CHN-CVA10
OP244629	TY2016216-SX-CHN-CVA10	OP244645	TY2019440-SX-CHN-CVA10
OP244630	TY2016267-SX-CHN-CVA10	OP244646	TY2019462-SX-CHN-CVA10
OP244631	TY2016536-SX-CHN-CVA10	OP244647	TY2019534-SX-CHN-CVA10
OP244632	TY2018007-SX-CHN-CVA10	OP244648	TY2019543-SX-CHN-CVA10
OP244633	TY2018364-SX-CHN-CVA10	OP244649	TY2020207-SX-CHN-CVA10
OP244634	TY2018441-SX-CHN-CVA10		
